# Towards a National Longitudinal Tracking Framework of Health Graduate Practice Locations

**DOI:** 10.1111/ajr.70085

**Published:** 2025-09-08

**Authors:** Vincent L. Versace, Pam Harvey, Geoff Argus, Christine Howard, Leanne Brown

**Affiliations:** ^1^ Deakin Rural Health, School of Medicine Deakin University Warrnambool Victoria Australia; ^2^ Monash Rural Health Monash University Bendigo Victoria Australia; ^3^ Southern Queensland Rural Health (SQRH), Faculty of Health and Behavioural Science The University of Queensland Toowoomba Queensland Australia; ^4^ School of Psychology and Wellbeing University of Southern Queensland Toowoomba Queensland Australia; ^5^ Three Rivers Department of Rural Health Charles Sturt University Wagga Wagga New South Wales Australia; ^6^ Department of Rural Health University of Newcastle Tamworth New South Wales Australia

## Abstract

**Aims:**

Workforce maldistribution is a challenge to the equitable provision of healthcare in Australia. This *Commentary* details how a multi‐university, large‐scale, and growing data asset is positioned to contribute strategically and operationally to addressing national workforce priorities.

**Context:**

The Nursing and Allied Health Graduate Outcome Tracking (NAHGOT) study is a prospective longitudinal research project with a commitment to nationwide geographical coverage. NAHGOT links practice location data (the outcome) from the Australian Health Practitioner Regulation Agency (Ahpra) with university administrative records, such as admission and placement data (explanatory variables). NAHGOT also links external surveys and publicly available spatial data describing socio‐economic conditions and access to services. There are seven universities formally part of the collaboration, with five others in the process of joining. NAHGOT has established a framework including project governance, data linkage and management protocols, and a central repository for de‐identified data.

**Approach:**

The background of NAHGOT, the benefits to the Rural Health Multidisciplinary Training program, current limitations and challenges, and the case for scale‐up and future direction are described.

**Conclusion:**

Universities are uniquely positioned to lead graduate tracking as they have access to a suite of key explanatory variables not available elsewhere. Increasing the number of participating universities within NAHGOT is a priority, as is broadening the pool of disciplines beyond those covered by Ahpra. The geo‐enrichment of placement and practice data is also a priority. This will allow a more granular understanding of local workforce dynamics that can be overlooked if analysis is limited to existing geographical classifications.

## 1 Background

Workforce maldistribution is an ongoing challenge in rural Australia, and addressing this priority is central to the guiding ambition of building fit‐for‐purpose health services to meet community need [1]. In response, the Department of Health, Disability and Ageing invests in the Rural Health Multidisciplinary Training (RHMT) program [2]. The program funds a network of University Departments of Rural Health (UDRH), Rural Clinical Schools (RCS), Regional Training Hubs (RTH), targeted rural medical programmes, and dental training sites. Of Australia's 43 accredited universities, 22 receive RHMT funding, with 16 RHMT‐funded universities involved in the delivery of a UDRH. University Departments of Rural Health are academic centres based in rural Australia, with a focus on health professional education and research, with a specific emphasis on nursing and allied health disciplines [2, 3, 4].

The RHMT has an overall program goal of improving the recruitment and retention of medical, nursing, dental, and allied health professionals in rural and remote Australia [5]. Achieving progress towards the program goal in a complex system requires a variety of levers within the delivery model. Activities within the implementation of the RHMT program include, but are not limited to, providing effective rural training opportunities, exploring and testing the efficacy of rural training settings, and increasing the number of students of rural origin. To aid in the delivery of predominantly place‐based activities, the RHMT program also includes a provision to ensure that funds are invested in the communities where program outcomes are targeted and has restrictions around expenditure in metropolitan areas [5]. Beyond the specific targets within the reporting framework, it is anticipated that the successful implementation of the RHMT will also see flow‐on benefits to the community through increased delivery of health services via student placement training activities. The enhancement of local health ecosystems as local clinical and academic leadership evolves in concert with long‐term investment is also anticipated [5].

Another area that has benefitted from long‐term RHMT investment is that of rural‐focused research—specifically driven by *Parameter 5: Maintaining and progressing an evidence base and the rural health agenda* [5]. A formal commitment to research within the policy framework drives activity in an area that would likely be overlooked at the national level given most of Australia's university campuses are based in metropolitan areas. Parameter 5 is focused on the outcomes embedded in the RHMT program aiding justification of the substantial investment [6]. Within Parameter 5, there is a specific reference to the establishment of graduate tracking systems as follows:

Collect and Maintain Data on Rural Workforce Outcomes Resulting From Rural Training Activity Through the RHMT Program.

Establish tracking systems for graduates, or utilise national data collections such as the Medical Schools Outcomes Database and the Australian Health Practitioner Regulation Agency, with a regional focus aligned to each university's operations within their rural communities [5].

In response, the Nursing and Allied Health Graduate Outcome Tracking (NAHGOT) study was established in 2017 by the University of Newcastle and Monash University, with Deakin University joining in 2019. The collaboration has since grown and now formally includes seven of the 16 RHMT‐funded universities (University of Newcastle, Monash University, Deakin University, University of South Australia, Charles Sturt University, University of Queensland, and University of Southern Queensland) with representation in New South Wales, Victoria, South Australia, and Queensland. There are five other universities in the process of formalising membership that will extend reach into Western Australia, the Northern Territory, and Tasmania, as well as further strengthen representation in Queensland, Victoria, and South Australia (Curtin University, University of Tasmania, James Cook University, La Trobe University, and Flinders University).

NAHGOT is a policy‐focused research project with a prospective longitudinal design and a commitment to nationwide geographical coverage [7]. NAHGOT integrates annually updated principal place of practice (PPP) location data (the outcome variable) from the Australian Health Practitioner Regulation Agency (Ahpra) with university administrative records, such as admission data, and placement location and duration (explanatory variables), and draws upon the national Student Experience and Graduate Outcomes Surveys. Additionally, publicly available Socio‐Economic Indexes for Areas (SEIFA) [8], a general measure of relative access to services (Australian Statistical Geography Standard Remoteness Areas, ASGS‐RA) [9], and a workforce‐specific measure of access (Modified Monash Model, MMM) [10] are appended to each graduate record—the latter being the current instrument used to define rural areas in the RHMT agreement [11]. Participating universities provide data conforming to an agreed format protocol to the secure central de‐identified data repository (hosted by Deakin University). The central repository is crucial to the collaboration as it allows seamless multi‐university studies across multiple disciplines and geographies to be undertaken.

All activity is covered by a formal Collaborative Research Agreement (CRA) and deed of admission (establishment led by University of Newcastle), and an active human research ethics approval (Monash University Human Research Ethics Committee, project identification 35528). Signing the CRA confers university support for NAHGOT, not just the UDRH—an important distinction that positions NAHGOT strategically as a national data asset. University‐level support means that *all* health professional graduates who register with Aphra will have a record in the central repository. This confers a database with much more end‐user utility, not only limited to rural workforce issues, but also able to respond to broader policy questions of national significance.

Beyond the RHMT, NAHGOT is aligned with the Stronger Rural Health Strategy (SRHS)—a ten‐year national strategy that commenced in 2018/19. The SRHS comprises three key themes: (1) teach, (2) train, and (3) recruit and retain. Within the SRHS, the expansion of the RHMT is explicitly referenced under the teaching theme, with a commitment to increased rural placements for health students [12].

## 2 Benefits to the RHMT

Many of the activities described and implemented within the parameters of the overall RHMT program framework are necessarily place‐based and respond to the heterogeneity of settings across rural Australia. This poses some challenges for the UDRH network in the development of generalisable models and collaboration more broadly. In recent times there has been a concerted effort to formalise efforts across the network, with the establishment and ongoing maintenance of the UDRH publication database providing a focal point for reviews into research outputs [13, 14, 15]. Definitionally, outcomes of this type are secondary research as they explore and synthesise existing primary research to better understand questions relevant to the UDRH network. NAHGOT complements the review work; the key difference being it positions the UDRH network to deliver primary research that is policy relevant and at scale [16, 17, 18].

NAHGOT has operational utility for applying data‐informed methods of matching students to placement opportunities during their studies (i.e., those with characteristics most likely to eventually practice in certain settings). This intelligence is of benefit to the university, local student placement teams, and the health service hosting the placement. Further to this, each participating university during the preparation of data for the NAHGOT central repository handles granular data (i.e., address level prior to categorisation as ASGS‐RA or MMM) that can be used to contribute to regional workforce strategies focused on the local communities they serve—a direct response to Parameter 5.

The overarching benefit of NAHGOT to the RHMT program is the increasing ability through a burgeoning membership to answer arguably the biggest question of all: *what is the impact of rural training on the eventual distribution of the workforce at scale over extended time periods?* Most studies of health professions graduate outcomes are cross‐sectional (i.e., a single time point outcome), with notable exceptions in the medical literature [e.g., 19, 20].

The first graduate data was entered into the NAHGOT database in 2017 and as time progresses and their annual PPP is updated, the evidence base for workforce outcomes will become stronger. In addition to being a research resource, NAHGOT also has the flexibility to evaluate long‐term program outcomes with end‐users across academic and policy domains. As an initiative, NAHGOT provides evidence of how the UDRH network can move beyond simply reporting annual core requirements and collaborate at scale to develop and deliver a nationally significant piece of e‐infrastructure.

## 3 Current Limitations and Challenges

NAHGOT partner universities comprise those that receive funding through the RHMT program and have voluntarily joined the collaboration. Part of the incentive for a university to join NAHGOT is that it provides an established framework to respond directly to graduate tracking aspects of Parameter 5. Although a majority of UDRH‐funded universities have formally joined or are in the process of joining (12/16), it is still a minority of all Australian universities (12/43). At this stage, there is no such policy incentive for non‐RHMT‐funded universities to join. Although many of the administrative aspects of NAHGOT are well established, ongoing resource allocation is required given the longitudinal design. Simply put, involvement with NAHGOT comes with an opportunity cost.

A core reporting requirement of the UDRHs is to annually report the number of clinical placement weeks that have been delivered, with overall targets differing between UDRHs. There is also a formal *additional requirement* embedded in the RHMT agreement that UDRHs provide placement opportunities to students from other universities. Currently estimates of placement activity across the network are limited to summing each UDRH's annual self‐reported total. This task is further complicated by overlapping geographies and a lack of a formal definition of what constitutes a clinical placement. A fully functioning NAHGOT central repository, assuming eventual full university participation, will provide the denominator of how many rural placements have been undertaken nationwide, acknowledging that there will be a time lag and will only come to light once graduation has occurred.

The structure of NAHGOT is currently limited to a selection of National Boards, who in conjunction with the National Agency (Ahpra), maintain public national registers of registered health practitioners. There are currently 15 National Boards, and it is where NAHGOT sources the primary outcome variable—the PPP. Eligibility to enter the NAHGOT central repository requires graduation and subsequent registration with Ahpra (currently, the seven university partners offer 11 Ahpra‐registered disciplines, Table 1). Key professions not covered by this arrangement include, but are not limited to, social workers and dietitians, professions considered to be self‐regulating through their respective professional associations (Table 1). A further complication of bringing new professions into NAHGOT is that practitioners of self‐regulated professions may not necessarily be members of their professional association.

**TABLE 1:** Summary of Ahpra and non‐Ahpra‐registered disciplines offered by the seven current members of the NAHGOT collaboration.

Discipline	MU	UoN	DU	UQ	UniSQ	UniSA	CSU
*Ahpra‐registered*							
Medical radiation practice	✓	✓	✓			✓	✓
Nursing	✓	✓	✓	✓	✓	✓	✓
Midwifery	✓	✓	✓	✓	✓	✓	✓
Occupational therapy	✓	✓	✓	✓	✓	✓	✓
Optometry			✓				
Oral health (oral/dental hygiene)		✓					✓
Paramedicine	✓				✓		✓
Pharmacy	✓	✓		✓		✓	✓
Physiotherapy	✓	✓		✓	✓	✓	✓
Podiatry	✓	✓				✓	✓
Psychology	✓		✓	✓	✓	✓	✓
*Non‐Ahpra‐registered*							
Audiology				✓			
Counselling				✓	✓		✓
Dietetics & nutrition	✓	✓	✓	✓		✓	
Exercise physiology			✓	✓	✓	✓	✓
Medical laboratory science					✓	✓	✓
Play therapy			✓				
Social work	✓		✓	✓	✓	✓	✓
Speech pathology		✓		✓	✓	✓	✓

Abbreviations: CSU, Charles Sturt University; DU, Deakin University; MU, Monash University; UniSA, University of South Australia; UniSQ, University of Southern Queensland; UoN, University of Newcastle; UQ, University of Queensland.

Within NAHGOT each university has responsibility for its own data management. In practical terms this means dealing with address‐level intelligence. The decision was taken early in the protocol development that the final data submitted to the central repository reporting PPP applied ASGS‐RA and the MMM—two measures that have been used over time to define rural within the RHMT [11]. While the ASGS‐RA and MMM can broadly inform what settings graduates practice in and eventually move between, they lack nuance. There are five ASGS‐RA classifications and seven MMM classifications, with much heterogeneity within classifications. As an example, while the NAHGOT central repository can be queried and report a de‐identified graduate practicing in MM5 in one year to the next, it cannot elucidate whether the student has moved towns, regions, or states within that classification. Another challenge in capturing the full impact of the RHMT at scale arises from the *additional requirement* described above that requires UDRHs to provide training opportunities to students from other universities. If support is given to a visiting student from a university not funded through RHMT, they have no way of being recorded in the NAHGOT central repository, even though the local UDRH can report on their activity through the routine RHMT core requirements template.

## 4 The Case for Scale‐Up and Future Directions

Solutions to several of the challenges and limitations described above are within reach and offer significant potential improvements to NAHGOT's overall utility as a national data asset. To date, membership of NAHGOT has been opt‐in and restricted to universities with a UDRH. Voluntary admission to NAHGOT remains open to the remaining universities within the UDRH network. As NAHGOT approaches saturation of universities that operate a UDRH, the time may be here when membership is broadened to all Australian accredited universities with relevant health course offerings. While ambitious, considering the number of universities and the number of disciplines, there is a precedent for this type of multi‐institutional level of cooperation—i.e., the Medical Deans Australia and New Zealand with membership comprising the 24 medical schools across Australia and New Zealand [21].

Follow‐up of health professions not covered by Ahpra remains an ongoing challenge (e.g., Table 1). NAHGOT has had informal discussions with the associations for several self‐regulating professions, recognising that it may not be a requirement for a health practitioner to be a member of their professional association, and each association will have its own unique member database and likely differing clauses for use of data for research purposes. For the foreseeable future, NAHGOT will be limited to those disciplines within the Ahpra framework. The advantage that NAHGOT offers is that participating universities are still able to internally process explanatory variables of non‐Ahpra disciplines in advance of future growth beyond Ahpra PPP.

Moving beyond reporting PPP, currenlty described as either ASGS‐RA or MMM, offers promise considering recent developments in the spatial description of health and education offerings [22]. Key examples include applying address‐level intelligence for the Regional Education Commissioner's 2023 Annual Report [23, 9–11] and a recent editorial that used distance/time from points contained in the Geocoded National Address File (GNAF) along the national road network to various educational locations (i.e., university campuses and Regional University Study Hubs) [24]. The same approach was used in the review of the MMM that formed part of the Working Better for Medicare Review [25, 90]. This work highlighted addresses that either had improved or reduced access to a general practice between 2015 and 2023. Address‐level intelligence has applications within NAHGOT to objectively measure distance from a graduate's residence at the time of enrolment to the campus where they studied, to their placements, and ultimately to where they practice. This process of geo‐enrichment would see privacy protected as this data is held at the university level. Only de‐identified spatial statistics would be appended to each record prior to submission to the central repository in the same way the existing publicly available spatial data is currently being appended (e.g., ASGS‐RA, MMM, SEIFA).

## 5 Conclusion

UDRHs are placed within university structures in part to ensure rural communities may gain from the broader societal benefits of these institutions, such as increased educational pathways and social capital. Although there are core requirements of the RHMT that are not directly aligned with traditional university business, NAHGOT offers a bridge that has a clear line of sight between the policy outcomes sought through the RHMT and the generation of high‐quality research outputs that universities value. Universities are the logical choice to undertake tracking of graduate outcomes at scale. Uniquely positioned as data custodians, universities hold admission and curriculum placement data not otherwise available.

To assist with scale‐up and to implement some of the identified future directions, a designated allocation of funding within the RHMT is required to provide certainty to NAHGOT in the first instance. As the project has grown, so has the administrative burden and associated costs, such as maintenance of the central repository. Involving non‐RHMT universities would require a further funding commitment, as would engagement with non‐Ahpra‐regulated professions. The structures in place will ensure value for money for this targeted investment. The framework that has been established by NAHGOT will allow for a non‐ambit estimate of what the establishment and maintenance costs would be with defined outcomes that would be guided by the CRA, the comprehensive data dictionary and central repository requirements, and the approved ethics.

Although database access is currently restricted to participating institutions, the longer‐term intent is to permit external data extractions through a formal process, including relevant ethics, to ensure NAHGOT reaches its potential as a national data asset for workforce planning in Australia over decadal time horizons.

## Author Contributions

This *Commentary* has been prepared by members of the Steering Group of the Nursing and Allied Health Graduate Outcome Tracking study (NAHGOT). All authors are Directors of University Departments of Rural Health at their respective institutions (VLV, Deakin University; PH, Monash University; GA, University of Queensland and University of Southern Queensland; CH, Charles Sturt University; LB, University of Newcastle). Collectively, the authors have wide experience in many of the challenges in the field of rural health, including training and retaining the workforce. Each author has contributed to the conception and writing of this *Commentary* as it draws upon their experience over a number of years setting the strategic direction of the NAHGOT study.

## Conflicts of Interest

The authors declare no conflicts of interest.

## Data Availability

The data that support the findings of this study are available from the corresponding author upon reasonable request.
